# Genomic selection on breeding time in a wild bird population

**DOI:** 10.1002/evl3.103

**Published:** 2019-03-05

**Authors:** Phillip Gienapp, Mario P. L. Calus, Veronika N. Laine, Marcel E. Visser

**Affiliations:** ^1^ Department of Animal Ecology Netherlands Institute of Ecology (NIOO‐KNAW) Wageningen The Netherlands; ^2^ Animal Breeding and Genomics Wageningen University & Research Wageningen The Netherlands

**Keywords:** Animal model, GBLUP, genomic breeding values, phenology, quantitative genetic

## Abstract

Artificial selection experiments are a powerful tool in evolutionary biology. Selecting individuals based on multimarker genotypes (genomic selection) has several advantages over phenotype‐based selection but has, so far, seen very limited use outside animal and plant breeding. Genomic selection depends on the markers tagging the causal loci that underlie the selected trait. Because the number of necessary markers depends, among other factors, on effective population size, genomic selection may be in practice not feasible in wild populations as most wild populations have much higher effective population sizes than domesticated populations. However, the current possibilities of cost‐effective high‐throughput genotyping could overcome this limitation and thereby make it possible to apply genomic selection also in wild populations. Using a unique dataset of about 2000 wild great tits (*Parus major*), a small passerine bird, genotyped on a 650 k SNP chip we calculated genomic breeding values for egg‐laying date using the so‐called GBLUP approach. In this approach, the pedigree‐based relatedness matrix of an “animal model,” a special form of the mixed model, is replaced by a marker‐based relatedness matrix. Using the marker‐based relatedness matrix, the model seemed better able to disentangle genetic and permanent environmental effects. We calculated the accuracy of genomic breeding values by correlating them to the phenotypes of individuals whose phenotypes were excluded from the analysis when estimating the genomic breeding values. The obtained accuracy was about 0.20, with very little effect of the used genomic relatedness estimator but a strong effect of the number of SNPs. The obtained accuracy is lower than typically seen in domesticated species but considerable for a trait with low heritability (∼0.2) as avian breeding time. Our results show that genomic selection is possible also in wild populations with potentially many applications, which we discuss here.

**Impact summary**

Selecting individuals based on their genotypes instead of their phenotypes is already widely applied in animal and plant breeding. This “genomic selection” has several advantages over “traditional” selection based on phenotypes, for example, individuals can be selected before they express their phenotype, which can considerably speed up selection in phenotypes that take a long time to measure or are difficult to obtain. Selection experiments can be a powerful tool in evolutionary biology and genomic selection could obviously be useful here. However, it has so far been unclear whether genomic selection will be feasible in natural populations that differ in important parameters, for example, effective population size, from populations of domestic breeds. We here explored whether genomic selection worked in a natural population of great tits (*Parus major*), a small, common songbird species. We could show that the accuracy, an important parameter determining the efficiency of genomic selection, of “genomic breeding values” for egg‐laying date in this population was moderate but lower than typical for animal and plant breeding. Despite this reduced accuracy, caused by the high effective population size of our large great tit study population, our results show that genomic selection can be possible in natural populations and we discuss a number of potential applications next to selection experiments.

Dissecting a trait's genetic architecture, i.e. how many (and also which) loci affect the trait and how their effect sizes are distributed, is important to fully understand and predict its evolution. Quantitative genetics assumes that a trait is determined by many loci of small effects. This assumption, the infinitesimal model (Barton et al. 2017), allows to model the traits’ genetic (co)variances and its evolutionary change, the response to selection, based on phenotypic resemblance among individuals of known relatedness without any molecular genetic information. Despite its unrealistic assumptions that, for example, totally ignore gene‐by‐gene interactions, for which quantitative genetics and the infinitesimal model have been criticized (Nelson et al. 2013), this framework has been highly successful in animal and plant breeding (Hill 2012) but also in natural populations (Charmantier et al. 2014).

The advances in molecular genetic techniques have led to increasing information about the molecular genetic architecture of traits. Gene mapping studies have been highly successful in mapping the genes underlying a variety of traits in humans (Visscher et al. 2017) but also other traits even in natural populations, as, for example, bill morphology in Darwin finches (Abzhanov et al. 2004, 2006), defensive armament traits in sticklebacks (Colosimo et al. 2004) or life‐history in salmon (Barson et al. 2015). Such gene mapping studies have, however, not been universally successful. In many cases, the identified causal loci could only explain a small part of the phenotypic variance (Manolio et al. 2009). This problem of “missing heritability” is now mostly resolved by an increasing number of markers included in the analyses (Visscher et al. 2017). The advances in molecular genetic techniques have now made it possible to undertake gene mapping studies in wild populations of “nonmodel” species. Unsurprisingly, however, these studies had limited power and often failed to identify any locus associated with the analyzed traits (see Supplementary Table in Gienapp et al. 2017b) indicating that many traits likely have a polygenic architecture. It hence seems that, despite its simplistic and not necessarily realistic assumptions (Nelson et al. 2013), the infinitesimal model of classical quantitative genetic theory is a reasonable approximation for the genetic architecture of many traits.

Quantitative genetics allows the prediction of individual “breeding values,” that is the sum of the additive genetic effects on the trait (Lynch and Walsh 1998; Mrode 2014), which are commonly used to select individuals in animal breeding but also allow testing for genetic differentiation, with some caveats (Hadfield et al. 2010), in space or time in natural populations (e.g., Garant et al. 2004; Gienapp and Merilä 2014). Quantitative genetic analysis requires information about relatedness among individuals, for example, from a pedigree. Establishing a pedigree from observational field work data is, however, only feasible in taxa with parental care where individuals can be individually and uniquely marked at the age when parental care still occurs. This means that quantitative genetic studies are generally biased toward certain bird and mammal species (Charmantier et al. 2014). An alternative, proposed already some time ago (e.g., Ritland 1996), is to use molecular markers to estimate relatedness among individuals instead of pedigrees. Early applications of this approach generally suffered from the limited number of markers that were available (Coltman 2005; Garant and Kruuk 2005) but using the currently available high‐throughput genotyping in “nonmodel” species may overcome this problem (Gienapp et al. 2017a).

In domesticated species high‐density marker panels have been available for quite some time. Starting with the influential paper by Meuwissen et al. (2001) the marker‐based prediction of breeding values, called “genomic selection” or “genomic prediction,” became a very widely accepted and applied tool in animal and plant breeding (Jannink et al. 2010; Meuwissen et al. 2016). “Genomic” breeding values (GEBVs), i.e. breeding values predicted from high‐density markers, are generally more accurate than pedigree‐based breeding values (Meuwissen et al. 2016). Furthermore, GEBVs have the advantage that they allow reasonably accurate predictions about the performance of individuals in the absence of own phenotypes (or offspring phenotypes in case of traits expressed only in the other sex). Genomic prediction relies on that the markers “tag” a sufficiently large number of the loci that determine the trait, that is each QTL (quantitative trait locus) is in linkage disequilibrium with at least one marker. This means that the accuracy of GEBVs depends, among other variables, on the number of phenotypic records and the extent of linkage disequilibrium that can be reflected by the number of independently segregating chromosome segments (*M_e_*) (e.g., Goddard 2009; Daetwyler et al. 2010).

The development of the necessary genomic tools for genomic selection in non‐domesticated species has generally been lagging behind but now high‐throughput high‐density genotyping has become possible in virtually any species. This potentially widens the scope of quantitative genetic studies in wild populations by allowing to use marker‐based relatedness instead of pedigree‐based relatedness and could enable us also to apply genomic selection in non‐domesticated species (Gienapp et al. 2017a). As pointed out above, the accuracy of GEBVs, and hence the feasibility of this approach, depends on *M_e_*, which in turns depends on the effective population size (*N_e_*) (e.g., Visscher et al. 2006). The effective population size is, however, generally much larger in natural than in domestic populations due to, for example, larger population census sizes, different mating systems and less skewed reproductive success. This will impair the feasibility of this approach and, so far, very few studies successfully predicted GEBVs in wild populations (e.g., Beaulieu et al. 2014).

We here applied genomic selection on seasonal breeding time in a wild population of great tits (*Parus major*). Knowledge about a trait's genetic architecture is also important to understand and predict its evolutionary response to selection. Climate change is generally expected to lead to selection on phenology (Gienapp et al. 2014) and it has been shown to lead to selection on phenology by disrupting synchrony between trophic levels in great tits (Visser et al. 1998) and other species (Visser and Both 2005). Whether populations or species will be able to successfully respond to selection depends, among other factors, on the genetic variance of the trait under selection (e.g., Lynch and Lande 1993; Bürger and Lynch 1995; Gienapp et al. 2013a). Avian breeding time is heritable in great tits (e.g., McCleery et al. 2004; Gienapp et al. 2006) but it is currently unclear which part of the “physiological cascade” underlying this trait varies genetically. By creating individuals with extreme phenotypes and studying their physiology we hope to be able to address this question. To create these extreme individuals we applied genomic selection in laboratory selection lines of great tits.

## Methods

### PHENOTYPES/TRAINING POPULATION

Our “training population,” that is the set of individuals for which phenotypes and genotypes are recorded, consisted of several long‐term study populations of wild great tits (*Parus major*) in the Southern part of the Veluwe area close to Arnhem (52° 00′ N, 5° 50′ E, the Netherlands). These populations are located in close vicinity (max. distance: 5 km) within a large contiguous woodland area. Great tits are small passerines that breed in natural cavities but readily accept artificial nest boxes. In all study populations nest boxes are supplied in overabundance so that almost all great tits breed in the supplied nest boxes. Nest‐boxes are checked weekly for signs of nest building and clutch initiation starting in the beginning of April. When a nest with eggs is found, the date of the first egg laid (hereafter egg‐laying date) is back‐calculated on the assumption that one egg is laid per day. All nestlings are ringed with standard aluminium bird rings at an age of seven days. Adults are caught in the nest boxes during the chick feeding period and identified by their rings or ringed if still unringed. This allowed the construction of a pedigree based on this observational data. In the recent decades adults and chicks have also been blood‐sampled at capture. Blood samples were stored in either 1 mL Cell Lysis Solution (Gentra Puregene Kit, Qiagen, USA) or Queens buffer (Seutin et al. 1991).

### GENOTYPING AND QUALITY CONTROL

A total of 2015 female great tits were genotyped using a custom made Affymetrix great tit 650K SNP chip (Kim et al. 2018) at Edinburgh Genomics (Edinburgh, United Kingdom). Axiom Analysis Suite 1.1 was used for SNP calling following the Affymetrix best practices workflow. Total of 32,716 SNPs located on unassigned reads, that is without known genomic position, and on the Z chromosome were excluded. Altogether 503,199 SNPs passed initial quality control. From these, 248 nonpolymorphic SNPs were excluded. SNPs were not filtered for Hardy–Weinberg equilibrium or minor allele frequency.

### CALCULATION OF GEBVs

There are two conceptually different ways to calculate genomic breeding values (GEBVs). In so‐called “whole genome regression” phenotypes are regressed simultaneously against all markers and the GEBV is calculated as the sum of all estimated marker effects multiplied with the corresponding genotypes (de los Campos et al. 2013). However, the fact that the number of estimated parameters, the marker effects, exceeds the number of observations prevents the use of “simple” least‐squares multiple regression and instead variable selection or shrinkage estimation procedures need to be used. Various approaches as, for example, ridge regression (Hoerl and Kennard 1970), LASSO (Tibshirani 1996) or Bayesian methods with various priors (e.g., Gianola 2013) have been proposed and used. The other approach is replacing the pedigree‐based relatedness matrix in an animal model by the marker‐based relatedness matrix (VanRaden 2008; Yang et al. 2010), generally known as GBLUP. While conceptually very different, this approach can be shown to be mathematically identical to ridge regression or a Bayesian approach with a Gaussian distribution of marker effects. Generally, the accuracies of GEBVs that can be obtained with these different approaches are comparable (e.g., Hayes et al. 2009a; Daetwyler et al. 2010; Gao et al. 2013).

We here calculated GEBVs using the GBLUP approach. The marker‐based or “genomic” relatedness matrix (GRM) was calculated using the pairwise relatedness estimators of VanRaden (2008), method 1, and Yang et al. (2010) using the program calc_grm (Calus and Vandenplas 2016). When using the option VanRaden 1, the GRM is calculated following VanRaden (2008) as

$$G=\frac{{\mathrm{ZZ}}^{\prime }}{2\sum p_{i}\left(1-p_{i}\right)}\;$$

with ***G*** being the GRM, ***Z*** being a centered matrix of marker genotypes of all individuals, and *p_i_* the frequency of the second allele at locus *i*. ***Z*** is calculated from the matrix of marker genotypes, coded as –1, 0, 1 for the homozygote, heterozygote, and other homozygote, by subtracting 2(*p_i_* – 0.5). Dividing ***ZZ***′ by $2\sum p_{i}\left(1-p_{i}\right)$ scales ***G*** to be analogous to the relatedness matrix obtained from a pedigree.

When using the option Yang, the GRM is calculated following Yang et al. (2010) as

$$G=\frac{{\mathrm{WW}}^{\prime }}{n}\;$$

with ***G*** being the GRM, ***W*** being a matrix containing the scaled and centered marker genotypes and *n* being the number of markers. The elements of ***W*** are computed as

$$\;w_{ij}=\frac{x_{ij}-2p_{i}}{\sqrt{2p_{i}\left(1-p_{i}\right)}}\;$$

with *x_ij_* being the marker genotype of individual *j* at locus *i*.

When a pedigree is known calc_grm offers the option to scale the GRM to the level of inbreeding in the pedigree following Powell et al. (2010). Finally, the GRM is adjusted for sampling error in the relatedness due to the limited number of markers following Yang et al. (2010). We here calculated the GRM with and without scaling according to the pedigree.

To explore the relative importance of relationship information contained in the pedigree and in the genomic relatedness matrix, we calculated a “weighted” (genomic) relatedness matrix ***G*** ^*^ as

$$G^{*}=G\alpha +A\left(1-\alpha \right)$$

with ***G*** being the GRM, ***A*** being the pedigree‐based relatedness matrix and α the weighting factor ranging from 0 to 1. Here, we used values for α of 0.05, 0.5, 0.8, and 0.95.

Because egg‐laying date is affected by spring temperature (e.g., Gienapp et al. 2005) and can also differ among habitats, we fitted the following model to all recorded egg‐laying dates and used the year and area estimates from this model to “precorrect” the recorded phenotypes of the genotyped individuals:

$$y_{i,j}=\mu +yr_{j}+ar_{a}+{\mathrm{age}}_{i}+{\mathrm{ind}}_{i}+\varepsilon$$

with *y_i,j_* being the phenotype of individual *i* in year *j*, *μ* the overall intercept, *yr_j_* and *ar_a_* the fixed effects for year (as factor) and area, respectively, *age_i_* the age of individual *i* (as factor, 1st year breeder vs older) and *ind_i_* the random effect of individual *i*. We did this, instead of fitting year and area in our GBLUP model, because not all individuals in all years were genotyped, which could have led to biases in the estimates for year‐area combinations with few genotyped individuals.

To estimate variance components and predict the GEBV at the same time we ran the following mixed model, our GBLUP model, in ASReml‐R:

$$y{}_{i,j}^{\prime }=\mu +{\mathrm{age}}_{i}+pe_{i}+a_{i}+\varepsilon$$

with *y′_i,j_* being the pre‐corrected phenotype of individual *i* in year *j*, that is $y{}_{i,j}^{\prime }=y_{i,j}\;-{\hat{yr}}_{j}-{\hat{ar}}_{a}$, *age_i_* the age of individual *i*, *pe_i_* the nongenetic (permanent environment) random effect of individual *i*, and *a_i_* the additive genetic random effect of individual *i*. The covariance between the additive genetic effects was given by the GRM or the weighted GRM (***G*** ^*^). For comparison, we also fitted a pedigree‐based model where the covariance between the additive genetic effects was given the pedigree‐based relatedness matrix, while the model was otherwise identical. The scripts to calculate the GRM and to run the animal model are available as Supplementary Material.

### VALIDATION OF GEBVs

In the “validation” step a subset of individuals that have both phenotypes and genotypes are excluded from the training population, and their precorrected phenotypes (see above) are regressed against the GEBVs predicted from their genotypes. The correlation between the GEBVs and phenotypes of these individuals is used to compute the accuracy of the GEBVs, which is defined as the correlation between the true and the estimated breeding values. Since the true breeding values are not known, phenotypes (P) that were precorrected for all fixed effects and averaged per individual are used instead:

$$\mathrm{accuracy}=\frac{cor(P,\;\mathrm{GEBV})}{\sqrt{h^{2}}}$$

where division by $\sqrt{h^{2}}$ corrects for the fact that the maximum correlation between P and GEBV is equal to $\sqrt{h^{2}}$, which is achieved if the accuracy is 1, that is when GEBV are equal to the true breeding values. To allow comparison of the accuracy obtained using different numbers of SNPs we used the *h* ^2^ obtained from the VanRaden model with all SNPs for scaling. For this comparison, we could hence equally well have used the correlation between phenotype and GEBV but choose to present the (identically scaled) accuracies to minimize potential confusion.

Since the standard error of the calculated accuracy depends on the size of the set of individuals excluded from the training population (Daetwyler et al. 2013), we followed a modified “leave‐one‐out” approach. In this approach each individual is in turn excluded from the training population, that is in practice its phenotype is set to missing and the standard GBLUP model is run. This means that this individual's GEBV is predicted based on all phenotypes except its own. To reduce computation time, we here did not exclude each individual separately from the training population but instead excluded 20 individuals at once, which reduced computation time by a factor 20. We randomly excluded 20 individuals from the training population, predicted their GEBVs and then selected the next 20 individuals (each individual was only excluded once) until all individuals were excluded once and their GEBVs predicted. When GEBVs for all individuals had been predicted, the GEBVs were correlated with the individuals’ phenotypes. 95% confidence intervals were obtained by bootstrapping. The 1000 bootstrap samples were obtained by randomly drawing—with replacement—2015 observations from the data. We also report the (approximate) standard errors of the accuracy and the root mean square error (RMSE) of the GEBVs. The standard error was based on the sampling variance of a correlation coefficient calculated as the square root of the sampling variance divided by the square root of the heritability. The RMSE was calculated as the square root of the mean squared difference between an individual's phenotype and its GEBV estimated including the individual's phenotype. The scripts that were used to run the validation analyses are available as Supplementary Material.

Since our data are from a natural population, there is some relatedness structure in the sampled individuals. Out of the 2015 individuals included in the analysis more than half (1520) had no known relatives within the genotyped individuals. This may be partly due to parents not being captured but overall capture rates of breeding adult individuals are at least 90%. Based on the recorded pedigree information, there were 185 individuals related as full‐sibs, 79 individuals related as maternal half‐sibs and 80 individuals related as paternal half‐sibs in the pedigree. Overall mean pairwise pedigree‐based relatedness was 0.00033.

To test the effect of the number of SNPs used to estimate relatedness we reran our analysis for subsets of 1000, 50,000, 100,000, 250,000, and 400,000 SNPs. For 100 replicates the corresponding number of SNPs was sampled randomly from the total number of SNPs, the GRM calculated using the VanRaden‐estimator, and GEBVs predicted using the approach described above for each random sample. That is, for the first replicate the phenotypes of the first 20 individuals were excluded and their GEBVs predicted, for the second replicate the phenotypes of the next 20 individuals were excluded and their GEBVs predicted and so forth.

### EXPECTED ACCURACY

We also calculated the expected accuracy of the GEBVs, following Daetwyler et al. (2010):

$$r_{g,\hat{g}}=\sqrt{\frac{Nh^{2}}{Nh^{2}+M_{e}}}\;$$

with *N* being size of the training population, *h* ^2^ heritability of the trait, and *M_e_* the number of independently segregating genome segments. We approximated *M_e_* using the equation of Goddard (2009):

$$M_{e}=\frac{2N_{e}L}{ln(4N_{e}L)}\;$$

with *N_e_* being effective population size and *L* genome length in Morgans. The total map length in the great tit study population is 2009.85 cM (van Oers et al. 2014). The effective population size estimated from pairwise sequential Markovian coalescent analysis is ∼5.7 × 10^5^ individuals (Laine et al. 2016). We also computed *M_e_* empirically from our data following Goddard et al. (2011):

$$M_{e}=\frac{1}{var(G)}\;$$

with *var*(*G*) being the variance across all off‐diagonal relationships in the GRM.

## Results

**Figure 1:**
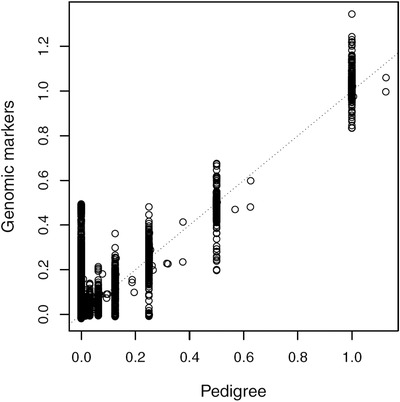
Correspondence between pairwise relatedness estimates from the pedigree and SNPs. The genomic relatedness estimated using all SNPs and the vanRaden (2008) estimator (including pedigree information, see Methods for details) is plotted against the relatedness estimated from the pedigree information. The dotted line indicates the 1:1 relationship.

The pairwise relatedness estimated from the pedigree and the GRM corresponded well (Fig. 1). The mean relatedness estimated from all SNPs, using VanRaden (2008) and scaled according to the pedigree (see Methods) for individuals with a pedigree relatedness of 0.5 (full sibs and parent‐offspring pairs) and 0.25 (half‐sibs in our dataset) was 0.49 and 0.22, respectively. A number of individuals that is unrelated according to the pedigree show “genomic” relatedness of up to 0.5. We checked these cases of unexpectedly high relatedness against our extensive data base and concluded that these could potentially be “missing links” in the pedigree, i.e. these individuals having a close common ancestor that is not recorded in the pedigree.

The heritabilities of egg‐laying date calculated from the different GRMs were consistently, although not significantly, lower than the estimate based on pedigree information (Table 1). At the same time, the permanent environment variance was considerably lower in the model using pedigree‐based relatedness. When the contribution of the GRM and pedigree to the relatedness estimates used in the analysis was varied, the results were very similar. The estimated heritability was highest when most weight was given to the GRM and decreased with decreasing weight of the GRM (Table 2).

**Table 1:** Estimates (and SE) for the additive genetic (*V_A_*), permanent environment (*V_PE_*), and residual (*V*_res_) variance component, heritability (*h* ^2^) of egg‐laying date and accuracies of GEBVs from animal models with different relatedness estimators (Yang: following Yang et al. (2010), VanRaden: following VanRaden (2008), Yang‐ped: as Yang but scaled for pedigree, VanRaden‐ped, as VanRaden but scaled for pedigree)

Relatedness estimator	*V_A_*	*h* ^2^	*V_PE_*	*V*_res_	Accuracy	95% CI	RMSE
Pedigree	5.77 (1.72)	0.24 (0.07)	2.64 (1.71)	15.7 (0.58)	0.197 (0.045)	0.115–0.281	4.723
VanRaden	3.91 (1.31)	0.16 (0.05)	4.48 (1.38)	15.6 (0.58)	0.210 (0.055)	0.105–0.304	4.746
Yang	3.84 (1.31)	0.16 (0.05)	4.55 (1.38)	15.6 (0.58)	0.206 (0.055)	0.097–0.312	4.746
VanRaden‐ped	4.00 (1.33)	0.17 (0.06)	4.39 (1.39)	15.6 (0.58)	0.207 (0.054)	0.104–0.304	4.745
Yang‐ped	3.94 (1.33)	0.16 (0.06)	4.45 (1.39)	15.6 (0.58)	0.206 (0.055)	0.103–0.303	4.746

The 95% confidence intervals of the accuracy as well as their root mean square error (RMSE) are also given.

**Table 2:** Estimates (and SE) for the additive genetic (*V_A_*), permanent environment (*V_PE_*), and residual (*V*_res_) variance component, heritability (*h* ^2^) of egg‐laying date and accuracies of GEBVs from animal models weighting pedigree and marker‐based relatedness to a different degree

Alpha	*V_A_*	*h* ^2^	*V_PE_*	*V*_res_	Accuracy	95% CI	RMSE
0.05	5.87 (1.72)	0.24 (0.07)	2.55 (1.71)	15.6 (0.58)	0.199 (0.045)	0.117–0.281	4.723
0.50	5.53 (1.62)	0.23 (0.207)	2.87 (1.62)	15.6 (0.58)	0.207 (0.046)	0.120–0.286	4.731
0.80	4.64 (1.45)	0.19 (0.06)	3.75 (1.49)	15.6 (0.58)	0.209 (0.051)	0.119–0.300	4.739
0.95	4.16 (1.36)	0.17 (0.06)	4.23 (1.42)	15.6 (0.58)	0.209 (0.053)	0.109–0.312	4.744

The 95% confidence intervals of the accuracy as well as their root means square error (RMSE) are also given. A high value for alpha means a high weight for marker‐based relatedness and vice versa.

**Figure 2:**
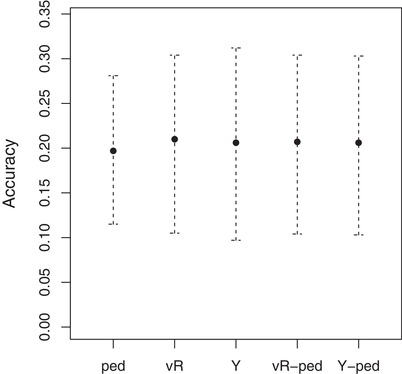
Estimated accuracies (and 95% confidence interval) for EBVs and GEBVs of egg‐laying date for four different estimation methods (ped: pedigree only, vR: vanRaden (2008), Y: Yang et al. (2010), vR‐ped: vanRaden (2008) including the pedigree in the estimation, Y‐ped: Yang et al. (2010) including the pedigree in the estimation). See Methods for details on estimation procedure.

The accuracies of (pedigree‐based) EBVs (Table 1) or of GEBVs with a strong contribution of the pedigree relative to the GRM (Table 2) were lower, but not substantially so, than accuracies based solely on the GRM. Which relatedness estimator, VanRaden (2008) or Yang et al. (Yang et al. 2010), was used and whether estimates were scaled by the pedigree had little effect on the accuracy of GEBVs (Fig. 2, Table 1). The root mean square errors (RMSEs) gave a slightly different picture with RMSEs being slightly lower for pedigree‐based analyses (Tables 1 and 2).

**Figure 3:**
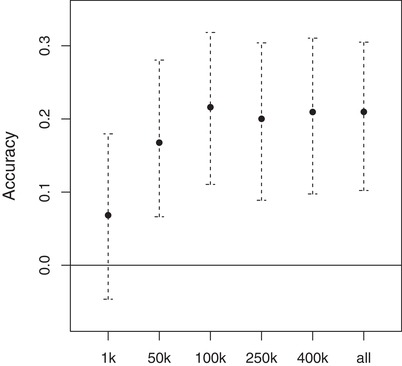
Accuracy of GEBVs (and 95% confidence interval) as a function of the number of markers used to estimate genomic relatedness (using VanRaden 2008).

The accuracy increased with the number of SNPs used to calculate the GRM (Fig. 3). Using only 1000 SNPs led to a very low accuracy, also with CIs overlapping with zero. Using 50,000 SNPs led to an improved but still reduced accuracy. Increasing the number of SNPs to 100,000 improved the accuracy still but whether 250,000, 400,000 or all SNPs were used made very little difference.

The predicted accuracy based on a heritability of 0.17, an effective population size of 570,000 individuals and a genome size of 20.1 M was much smaller than the empirical accuracy, namely only 0.017, when *M_e_* was calculated from *N_e_*, following Goddard (2009) giving a value of 1,298,858. When *M_e_* was calculated empirically from the variation in the relatedness, following Goddard et al. (2011) giving a value of 7230, the predicted accuracy was 0.21 and thus very close to the empirical accuracy.

## Discussion

Depending on the used relatedness estimator and whether estimates were scaled according to the pedigree the estimated accuracy of GEBVs for egg‐laying date varied between 0.197 and 0.210. This is lower than what is normally found in domesticated species. One study in dairy cattle on a variety of traits found accuracies that ranged from 0.63 to 0.83, with an average of 0.70 (VanRaden et al. 2009). An early review study on dairy cattle based on considerably smaller training populations of only 332 to 637 individuals still found accuracies of 0.60 on average (Hayes et al. 2009b). The average accuracy in a large number of studies on plants, mainly crop species, was ca. 0.60 (reviewed in Lin et al. 2014). It hence seems that the accuracies estimated here are smaller than the ones typically found in domesticated animal and plant species. This is maybe not too surprising as genomic prediction relies on the markers being in linkage disequilibrium with the loci determining the trait. Effective population sizes of cattle breeds can be as low as ∼100 (e.g., de Roos et al. 2008). Due to the larger effective population sizes of natural populations and the therefore higher number of independently segregating genome segments fewer causal loci will be “tagged” by the markers in natural populations resulting in lower expected accuracy of GEBVs (e.g., Goddard 2009; Hayes et al. 2009c; Daetwyler et al. 2010). Another factor determining the accuracy is the number of individuals in the training population, i.e. the number of individuals of which both phenotype and genotype are known. For example, increasing the size of the training population from 1151 to 3576 individuals increased the accuracy of the predicted GEBVs from 0.35 to 0.53 in Holstein dairy cattle (VanRaden et al. 2009). Our training population of about 2000 individuals was smaller than typical datasets from animal and plant breeding, especially recent studies that have been based on >20,000 genotyped individuals (e.g., Lin et al. 2014; Schöpke and Swalve 2016). The accuracy of GEBVs also depends on the trait's heritability (e.g., Goddard et al. 2011; Su et al. 2012; Brito et al. 2017), which was 0.24 when estimated from the pedigree and 0.17 when estimated using the markers and hence roughly comparable to the traits analyzed in, for example, VanRaden et al. (2009).

The predicted accuracy of the GEBVs for egg‐laying date was lower than the estimated accuracy and also very low in absolute terms, about 0.01, when estimating *M_e_* from the effective population size in Laine et al. (2016). The factor driving this low predicted accuracy was the very large effective population size in great tits, more than half a million (Laine et al. 2016). While there is very little genetic differentiation between European great tit populations (Kvist et al. 2003; Laine et al. 2016), this very large effective population size may still be an overestimate. When we calculated *M_e_* from the variation in genomic relatedness, the predicted and estimated accuracies corresponded much better (0.21 vs. 0.18). This illustrates that estimating *M_e_* from *N_e_* can be challenging (Brard and Ricard 2015).

Goddard et al. (2011) showed that the number of markers also affects the accuracy of GEBVs because if the number of markers is too small the true relatedness at the causal loci will be estimated too imprecisely. The expected accuracy increased with the number of SNPs but started to plateau at around 3000 SNPs (Goddard et al. 2011). This is in line with earlier findings that increasing the number of SNPs from ∼10,000 to ∼38,000 had very little effect on estimated accuracies in Holstein dairy cattle (VanRaden et al. 2009). We used here more than ten times more markers, 502,951 SNPs, which seemed to have “compensated” for the larger effective population size and the hence increased number of independently segregating genome segments. We also could demonstrate the previously expected pattern that the accuracy plateaued with an increasing number of markers indicating that the number of markers used here was sufficient (Fig. 3).

The accuracy of the estimated breeding values (EBVs) from a pedigree‐based animal model was very similar to the accuracies of GEBVs but the pedigree‐based heritability was higher (Table 1). The effect that using the pedigree rather than the GRM led to higher heritabilities could also be seen when more weight was given to the pedigree matrix relative to the GRM in a combined, weighted relatedness matrix (Table 2). One potential reason for the higher pedigree‐based estimate is environmentally caused similarity among relatives inflating estimates of *V_A_* and *h* ^2^ (van der Jeugd and McCleery 2002). Animal models have been thought to suitably account for this potential bias (e.g., Kruuk 2004; Wilson et al. 2010) but a simulation study showed that not all pedigrees, including the one of the Hoge Veluwe study population, may be informative enough to allow this (Gienapp et al. 2013b). The low relatedness information in the pedigree‐based analysis means that it is difficult for the model to disentangle the genetic and permanent environmental effects with too much of the permanent environment variance being absorbed into the additive genetic variance and the EBVs. Using genomic instead of pedigree relatedness has been shown to improve the ability of the model to disentangle variances of possibly confounded effects (Lee et al. 2010). Thus, the additional information contained in the GRM compared to the pedigree may have been able to remove more of the environmentally caused similarity among relatives, which would mean that the pedigree‐based heritability estimate would be inflated. However, to ultimately address this, cross‐fostering experiments (e.g., Kruuk and Hadfield 2007) or models directly fitting the spatial correlation would be necessary (e.g., Stopher et al. 2012).

While GEBVs have an obvious use in animal and plant breeding, they also have the potential for useful applications in natural populations. Whether this will be possible and useful obviously depends on the accuracy of the estimated GEBVs, which will depend on species‐ and population‐specific parameters, which determine *N_e_* and *M_e_*, and the number of available markers. Whether these potential constraints prohibit the useful applications of GEBVs in certain species or populations, requires further investigation. GEBVs could, for example, be used to select individuals for assisted migration or release programs. Assisted migration aims to mitigate negative effects of climate change by transplanting suitable individuals to new locations that could only be slowly reached by natural dispersal, that is “assisting” their dispersal, which has already been used in forest tree management (Aitken and Whitlock 2013). In breeding programs for endangered species GEBVs could also play a role by helping to identify individuals that are well adapted to the, potentially altered, release environments (Griffiths and Pavajeau 2008). Currently, genotyping is more costly than measuring phenotypes but with dropping genotyping costs this may change, especially for phenotypes that are difficult to measure. Even though the variance in estimated (genomic) breeding values is downward biased in comparison to the additive genetic variance (e.g., Hadfield et al. 2010) it may still serve as a useful proxy. Genotyping individuals in newly studied populations and predicting their GEBVs could allow us to (roughly) predict the evolutionary potential of these populations without the need for, potentially very laborious, quantitative genetic studies. Potential limitations of GEBVs for such applications can, however, arise from Genotype‐by‐Environment interactions (G × E) or different genetic trait architectures in different populations. G × E is common in many traits (e.g., Pigliucci 2001; Wood and Brodie 2016), which would mean that the “ranking” of individual breeding values in different environments can differ. As a consequence of this, the accuracy of predicting GEBVs across environments can be substantially reduced. Predicting GEBVs across populations, while ignoring this in the model, assumes that the genetic architecture of the trait is identical, that is that the same loci segregate in both populations. Cross‐breed prediction of GEBVs in domesticated populations has been attempted but accuracies are much lower than typical within‐breed accuracies (Moghaddar et al. 2014; Hidalgo et al. 2016).

High‐density marker panels have been available in animal and plant breeding and have been used in these fields for considerably longer than in evolutionary ecology to estimate relatedness from markers or even genomic selection (Gienapp et al. 2017a). Early approaches aimed at estimating relatedness in natural populations from markers failed due to the limited number of markers available at that time (Coltman 2005; Csilléry et al. 2006), which is again stressed by our results that about 100,000 SNPs were necessary to obtain reliable accuracies of GEBVs. More recently, studies in natural populations of mammals, birds, and plants successfully estimated additive genetic variances in a number of traits and were even able to predict GEBVs (Robinson et al. 2013; Stanton‐Geddes et al. 2013; Beaulieu et al. 2014; Bérénos et al. 2014). Consequently, genomic prediction and also marker‐based quantitative genetics could become possible in a wide range of species, which has the potential to widen our understanding of evolutionary dynamics in natural populations (Gienapp et al. 2017a).

Associate Editor: A. Charmantier

## Supporting information

Supplementary Material

## Data Availability

All data will be deposited in a public archive (Dryad) after acceptance and before publication of the manuscript.
