# Supplementary material for: Genomic selection on breeding time in a wild bird population
Source: Evol Lett. 2019 Mar 5;3(2):142–51. doi: 10.1002/evl3.103 (PMC6591552; doi:10.1002/evl3.103)
Supplement: Supplementary file 1 — Supplementary Material [file EVL3-3-142-s001.pdf]

Supplementary Material

1. Linux shell script and R code to run the GBLUP model

1.1 Re-format genotypes from standard PLINK format to format that can be read by calc_grm

```
plink --allow-extra-chr --chr-set 32 --bfile MY.GENOTYPES0 --recode A --out MY.GENOTYPES

cut -d " " -f 1,3,4,5,6 --complement MY.GENOTYPES.raw > MY.GENOTYPES.txt   # This removes 1st, 3rd, 4th and 5th column from the PLINK file

sed -i '1d' MY.GENOTYPES.txt                                            # This removes the first (header) row.

sed -i -e 's/NA/9/g' MY.GENOTYPES.txt                                   # This replaces missing genotypes coded ‘NA’ by ‘9’.
```

1.2 Run calc_grm by first creating .txt-files that tell calc_grm what to do and then calling calc_grm

Because the names of the outputted files cannot be set in calc_grm, they need to be renamed manually afterwards or stored in different directories.

```
echo """503199                       # number of SNPs

 MY.GENOTYPES.txt MY.PEDIGREE.txt    # identify genotype and pedigree files
                                     # If no pedigree should be fitted, do not specify pedigree file.

 genotypes                           # specify genotype format

 1

 vanraden                            # specify how GRM is calculated, alternative: ‘yang’

 giv                                 # specify that inverse of G should calculated
```

```
 G ASReml                              # specifcy output format of Ginv

 print_giv=asc

 print_geno=no

 1""" > calc_grm.inp

calc_grm                               # run calc_grm
```

1.3 R code to run GBLUP model

```
library(asreml)

d1 <- MY.DATA.txt                      # read in data file with 'CentredLD' being the pre-corrected egg-laying date, 'age' a two-level factor,
                                       # 'RingNumber' the individual's ring number for fitting the permanent environment effect, and 'SampleID' an
                                       # integer variable for individual to link to genotypes

d1$animal <- as.factor(as.character(d1$SampleID)     # create variable to genomic relatedness

giv <- read.table("G_asreml.giv")      # read in inverse of GRM in ASReml-format as created by calc_grm. Which GRMinv is read in here determines whether
                                       # the GBLUP model is run using the GRM calculated according to VanRaden1 or Yang and whether it is scaled to the
                                       # inbreeding level in the pedigree. So the correct name or path needs to be specified here.

names(giv) <- c('row','column','G','id1','id2')

giv1 <- giv[,1:3]                      # re-format to ASReml-R format

colnames(giv1) <- c('Row','Column','Ainverse')

attr(giv1,'rowNames') <- as.factor(unique(giv$id1))


am1 <- asreml(CentredLD~age, random=~ RingNumber + ped(animal,var=T), ginverse=list(animal=giv1), data=d1, workspace=32e6)
                                                                             # fit GBLUP model while increasing workspace size
```

2. R code to run the validation analysis, using all SNPs

2.1 R code that randomly selects individuals that are excluded from the training population, and runs the GBLUP model. It is assumed to be run after the GBLUP model with all observations (1.3) so data and GRMinv are still in the workspace.

```
rind <- read.table('inds_rand_order.txt')    # read in file where individuals are stored in random order to avoid excluding whole 'families' together.
                                             # To ensure that the same order is used in all validations the random order was stored in a file rather
                                             # rather than shuffling individuals a new for each analysis.

inds <- rind$V1               # vector of individuals in random order

for (i in 1:100 ) {           # cycle over first 2000 individuals

       id.miss.P <- inds[1:20+(i-1)*20]                              # determine the ith set of 20 individuals to be excluded

       d1$cLD <- ifelse(d1$SampleID%in%id.miss.P,NA,d1$CentredLD)  # set their phenotypes to missing

       am <- asreml(cLD~age, random=~ RingNumber + ped(animal,var=T), ginverse=list(animal=giv1), data=d1, workspace=32e6)     # run GBLUP model

       k1 <- data.frame(ind=row.names(coef(am)$random),coef(am)$random)           # store all BLUPs

       k1$animal <- as.numeric(substr(as.character(k1$ind),22,nchar(as.character(k1$ind))))

       k1 <- subset(k1,animal%in%id.miss.P & is.na(animal)==F)                                         # select GEBVs of excluded individuals

       d1$gebv <- ifelse(d1$SampleID%in%id.miss.P,k1$effect[match(d1$SampleID,k1$animal)],d1$gebv) }     # add their GEBVs to data

id.miss.P <- inds[2001:2015]          # do the same for the last remaining 15 individuals

d1$cLD <- ifelse(d1$SampleID%in%id.miss.P,NA,d1$CentredLD)

am <- asreml(cLD~age, random=~ RingNumber + ped(animal,var=T), ginverse=list(animal=giv1), data=d1, workspace=32e6)

k1 <- data.frame(ind=row.names(coef(am)$random),coef(am)$random)

k1$animal <- as.numeric(substr(as.character(k1$ind),22,nchar(as.character(k1$ind))))

k1 <- subset(k1,animal%in%id.miss.P & is.na(animal)==F)

d1$gebv <- ifelse(d1$SampleID%in%id.miss.P,k1$effect[match(d1$SampleID,k1$animal)],d1$gebv)
```

2. R code and Linux shell script to run the validation analysis for different numbers of SNPs

The R code randomly selects subsets of *n* markers, calls a Linux shell script that formats the genotypes and calculates the GRM based on this marker subset, and runs the GBLUP excluding each time 20 individuals from the training population to predict their GEBVs for the validation.

2.1 R code

```
### set number of SNPs in 1000

nsnps <- 100

d1 <- MY.DATA.txt                             # read in data file with 'CentredLD' being the pre-corrected egg-laying date, 'age' a two-level factor,
                                              # 'RingNumber' the individual's ring number for fitting the permanent environment effect, and 'SampleID'
                                              # an integer variable for individual to link to genotypes

d1$animal <- as.factor(as.character(d1$SampleID)     # create variable to genomic relatedness

rind <- read.table('inds_rand_order.txt')     # read in file where individuals are stored in random
                                              # order to avoid excluding whole 'families' together

inds <- rind$V1                               # vector of individuals in random order

d.snp <- read.table('snps.txt')               # read in file with SNP names


for (i in 1:100) {     # select randomly nsnps SNPs, calculate GRM based on these, run validation for 20 randomly selected individuals

       snp.sel <- sample(d.snp$snp,nsnps*1000)              # select random SNPs

       sel <- d.snp$snp%in%snp.sel

       d.snp <- data.frame(d.snp,sel) ; names(d.snp) <- c(names(d.snp)[1:(dim(d.snp)[2]-1)],paste('sel',i,sep='-'))

       write.table(snp.sel,paste('nsnps,'k_vR/selected_snps.txt',sep=''),row.names=F,col.names=F,quote=F)       # export selected SNPs

       system(paste('sh script_',nsnps,'k.sh',sep=''), wait=T) # run shell script to re-format SNP data and calculate GRM

       giv <- read.table(paste('nsnps,'k_vR/G_asreml.giv',sep=''))         # read in GRM
```

```
        names(giv) <- c('row','column','G','id1','id2')                     # re-format GRM
        giv1 <- giv[,1:3]
        colnames(giv1) <- c('Row','Column','Ainverse')
        attr(giv1,'rowNames') <- as.factor(unique(giv$id1))
        id.miss.P <- inds[1:20+(i-1)*20]
        d1$cLD <- ifelse(d1$SampleID%in%id.miss.P,NA,d1$CentredLD)
        am <- asreml(cLD~age, random=~ RingNumber + ped(animal,var=T), ginverse=list(animal=giv1), data=d1, workspace=32e6)
        k1 <- data.frame(ind=row.names(coef(am)$random),coef(am)$random)
        k1$animal <- as.numeric(substr(as.character(k1$ind),22,nchar(as.character(k1$ind))))
        k1 <- subset(k1,animal%in%id.miss.P & is.na(animal)==F)
        d1$gebv <- ifelse(d1$SampleID%in%id.miss.P,k1$effect[match(d1$SampleID,k1$animal)],d1$gebv) }

# select randomly nsnps SNPs, calculate GRM based on these, run validation for last 15 individuals
snp.sel <- sample(d.snp$snp,nsnps*1000)             # select random SNPs
sel <- d.snp$snp%in%snp.sel
d.snp <- data.frame(d.snp,sel) ; names(d.snp) <- c(names(d.snp)[1:(dim(d.snp)[2]-1)],paste('sel',i,sep='-'))
system(paste('sh script_',nsnps,'k.sh',sep=''), wait=T)
giv <- read.table(paste('nsnps,'k_vR/G_asreml.giv',sep=''))         # read in GRM
names(giv) <- c('row','column','G','id1','id2')                     # re-format GRM
giv1 <- giv[,1:3]
colnames(giv1) <- c('Row','Column','Ainverse')
attr(giv1,'rowNames') <- as.factor(unique(giv$id1))
id.miss.P <- inds[2001:2015]          # define missing individuals – last 15 individuals
d1$cLD <- ifelse(d1$SampleID%in%id.miss.P,NA,d1$CentredLD)
```

```
am <- asreml(cLD~age, random=~ RingNumber + ped(animal,var=T), ginverse=list(animal=giv1), data=d1, workspace=32e6)

k1 <- data.frame(ind=row.names(coef(am)$random),coef(am)$random)

k1$animal <- as.numeric(substr(as.character(k1$ind),22,nchar(as.character(k1$ind))))

k1 <- subset(k1,animal%in%id.miss.P & is.na(animal)==F)

d1$gebv <- ifelse(d1$SampleID%in%id.miss.P,k1$effect[match(d1$SampleID,k1$animal)],d1$gebv)
```

2.2 Linux shell script

Since the R script calls a specifically named shell script depending on the number of SNPs used, this script has to be adjusted and saved under the corresponding name.

```
### re-format genotypes 'for sharing' from PLINK format to genotype format for calc_grm, using randomly selected SNP subset

plink --allow-extra-chr --chr-set 32 --bfile MY.GENOTYPES0 --extract selected_snps.txt --recode A --out MY.GENOTYPES


cut -d " " -f 1,3,4,5,6 --complement MY.GENOTYPES.raw > MY.GENOTYPES.txt          # remove 1st, 3rd, 4th and 5th column

sed -i '1d' MY.GENOTYPES.txt                                                       # remove 1st row

sed -i -e 's/NA/9/g' MY.GENOTYPES.txt                                              # replace NAs by 9 as identifier for missing values


### calculate GRMs

echo """100000                # Here the number of SNPs need to be changed

 MY.GENOTYPES.txt

 genotypes

 1

 vanraden

 giv
```

```
 G ASReml
 print_giv=asc
 print_geno=no
 1""" > calc_grm.inp
calc_grm
```
